# Clinical Effect Size of an Educational Intervention in the Home and Compliance With Mobile Phone-Based Reminders for People Who Suffer From Stroke: Protocol of a Randomized Controlled Trial

**DOI:** 10.2196/resprot.4034

**Published:** 2015-03-10

**Authors:** Jose Antonio Merchán-Baeza, Manuel Gonzalez-Sanchez, Antonio Cuesta-Vargas

**Affiliations:** ^1^Universidad de MalagaMalagaSpain

**Keywords:** stroke, ADL, environment, patient adherence, mobile apps, mobile health

## Abstract

**Background:**

Stroke is the third-leading cause of death and the leading cause of long-term neurological disability in the world. Cognitive, communication, and physical weakness combined with environmental changes frequently cause changes in the roles, routines, and daily occupations of stroke sufferers. Educational intervention combines didactic and interactive intervention, which combines the best choices for teaching new behaviors since it involves the active participation of the patient in learning. Nowadays, there are many types of interventions or means to increase adherence to treatment.

**Objective:**

The aim of this study is to enable patients who have suffered stroke and been discharged to their homes to improve the performance of the activities of daily living (ADL) in their home environment, based on advice given by the therapist. A secondary aim is that these patients continue the treatment through a reminder app installed on their mobile phones.

**Methods:**

This study is a clinical randomized controlled trial. The total sample will consist of 80 adults who have suffered a stroke with moderate severity and who have been discharged to their homes in the 3 months prior to recruitment to the study. The following tests and scales will be used to measure the outcome variables: Barthel Index, the Functional Independence Measure, the Mini-Mental State Examination, the Canadian Neurological Scale, the Stroke Impact Scale-16, the Trunk Control Test, the Modified Rankin Scale, the Multidimensional Scale of Perceived Social Support, the Quality of Life Scale for Stroke, the Functional Reach Test, the Romberg Test, the Time Up and Go test, the Timed-Stands Test, a portable dynamometer, and a sociodemographic questionnaire. Descriptive analyses will include mean, standard deviation, and 95% confidence intervals of the values for each variable. The Kolmogov-Smirnov (KS) test and a 2x2 mixed-model analysis of variance (ANOVA) will be used. Intergroup effect sizes will be calculated (Cohen’s d).

**Results:**

Currently, the study is in the recruitment phase and implementation of the intervention has begun. The authors anticipate that during 2015 the following processes should be completed: recruitment, intervention, and data collection. It is expected that the analysis of all data and the first results should be available in early-to-mid 2016.

**Conclusions:**

An educational intervention based on therapeutic home advice and a reminder app has been developed by the authors with the intention that patients who have suffered stroke perform the ADL more easily and use their affected limbs more actively in the ADL. The use of reminders via mobile phone is proposed as an innovative tool to increase treatment adherence in this population.

**Trial Registration:**

ClinicalTrials.gov NCT01980641; https://clinicaltrials.gov/ct2/show/NCT01980641 (Archived by WebCite at http://www.webcitation.org/6WRWFmY6U).

## Introduction

Stroke is the third leading cause of death and the leading cause of long-term neurological disability in the world [1-3]. In Europe, 250 people per 100,000 suffer strokes each year, and this trend is worsening with time [1,4]. More than half of all patients who survive a stroke suffer a severe disability that causes limitations in their independent functioning and their performance of activities of daily living (ADL) [1,3-5]. The prevalence of stroke is around 2% in people over 20 years of age, increasing to 6 to 7% for those over 65. In men, 66.5% of strokes occur in people over 65 years old, while in women this percentage increases to 80.3% [6,7].

Researchers have examined the impact of stroke on patients who have suffered one and they have shown that cognitive, communication, and physical weakness combined with environmental changes frequently cause changes in roles, routines, and daily occupations [8-11].

Stroke patients often receive treatment from a multidisciplinary team, such as physiotherapy or occupational therapy, during their stay in hospital or in a rehabilitation service after home discharge [5,12-14]. There are even some cases in which stroke survivors receive aerobic and endurance training [15], strength, balance, and coordination training [16], a comprehensive geriatric intervention [17,18], or functional activity training [19] at home.

Another type of treatment is educational intervention, which is used in patients with different pathologies and even with professionals, thanks to its proven effectiveness [20-23]. Educational intervention combines didactic and interactive intervention [24], which combines the best choices for teaching new behaviors since it involves the active participation of the patient in learning [23]. The effectiveness of this intervention lies in the fact that patients synthesize and apply what they have learned, which is a reinforcement learning behavior [20,21]. In turn, the educational intervention can offer cost savings in the rehabilitation process because of the possible reduction in patient visits to their general practitioner, the emergency department, and/or specialists, as well as reduction in the use of drugs [25].

Some studies have shown the application of this type of intervention in people who have suffered a stroke as a means to reduce the risk of secondary stroke [26]. Educational intervention in this population remains unusual, however, despite being in great demand, due to the lack of information individuals often encounter in their rehabilitation process [26,27]. Therefore, in order to allow for continuity and applicability to the previous rehabilitation treatment received in the hospital, we propose an educational intervention at home for people who have suffered a stroke and who have been discharged to their homes in the 3 months prior to recruitment to the study. The therapist—using a tool for home therapeutic counselling—will perform an ergonomic assessment of the home and of the execution of the ADL by the participant. Subsequently, based on the items not presented ​​in the tool, he or she will provide advice on the correct or easiest way to perform ADL, on which adaptations they should make at home, and on what kind of technical assistance could be useful for them [28,29].

Patient satisfaction regarding information and knowledge about treatment are key to adherence to this therapy by patients with long-term diseases [30]. Nowadays, there are many types of interventions used to increase adherence to treatment, such as Web-based programs, video conferencing, or other means that are available due to technological advances [31,32].

Furthermore, mobile phones are increasingly being used in clinical practice to assess patients or for more precise tracking [32-35]. The development of mobile phone apps has favored diagnosis and early intervention in people who have suffered, or will suffer, a stroke [33-35]. Memory-aiding therapeutic apps have helped to improve the results of interventions for stroke patients as a result of the inclusion of the patients as an active part of the treatment [32].

Therefore, in this study we propose the development of a mobile phone app that acts as a daily reminder of the advice that was given to patients by the therapist during the educational intervention in their homes.

The aim of this study is that patients who have suffered stroke and have been discharged to their homes improve the performance of ADL in their home environments, following the restrictions caused by the stroke, after having been given advice by the therapist. A secondary aim is that these patients continue the treatment through a reminder app installed on their mobile phones (mHealth).

The hypothesis of this study is that patients who have suffered stroke will perform the ADL more easily and use their affected limbs more actively in the ADL after an educational intervention. The use of reminders via mobile phone is proposed as a tool to increase treatment adherence.

## Methods

### Design

This study is a clinical randomized controlled trial (RCT) and will be conducted following the CONSORT rules for reporting [36]. This trial has been registered with ClinicalTrials.gov (NCT01980641).

### Participants

The total sample will consist of 80 adults who have suffered a stroke with moderate severity (score between 0 and 49 on the Barthel Index [37]), who have been discharged to their homes in the 3 months prior to recruitment to the study [28]. The sample will be taken from the Carlos Haya Hospital complex in Málaga, Spain.

Individuals with dementia or other severe cognitive impairment (scoring 0 to 17 in the Mini-Mental State Examination) will not be included [38].

### Randomization

In stage 1, the sample will be divided into two groups of 40 participants each—experimental group and control group. The allocation and the randomization will be performed by a blinded researcher. The assignment of subjects to each group will be made through a system of sealed envelopes. Subsequently, for the pilot study in stage 2, we will create a group that will receive the app reminders on their mobile phones—the mobile phone group—and another group that will not receive the app reminder—the no mobile phone group. Group allocation will depend on whether the participant has a mobile phone and if its characteristics are adapted to the requirements of the study.

### Educational Intervention

The therapist will go to each participant's home and perform an ergonomic assessment of the home. The therapist will also assess the execution of ADL by the participant using the home therapeutic advice for people who suffer stroke (HTAS) tool, which is a checklist of 60 items—the therapist will mark those items he valued as deficient. Later, with the experimental group, the therapist will mark on the participant’s advice sheet those items that were rated negatively and will advise them on how to solve these shortcomings. With this advice, the therapist will educate participants on the correct or easiest way to perform their daily tasks, on which adaptations they should make at home, and on what kind of technical assistance could be useful for them. Previous studies on the impact of stroke in patients have demonstrated that cognitive, communication, and physical weakness combined with environmental changes frequently cause changes in roles, routines, and daily occupations [8-11].

In stage 2 of the study, the app will be installed on the mobile phones of participants in the mobile phone group, which will remind them of the advice previously offered by the therapist. The timing of reminders will differ for each participant depending on the amount of advice they received. However, in the 18 weeks of the app being used, each piece of advice will be given as a reminder three times. The mobile phone will beep once for each piece of advice and the participant must check and mark the option indicating whether he has complied with the advice or not. Participants who will take part in the app intervention will have to answer at least 80% of the messages.

**Figure 1:**
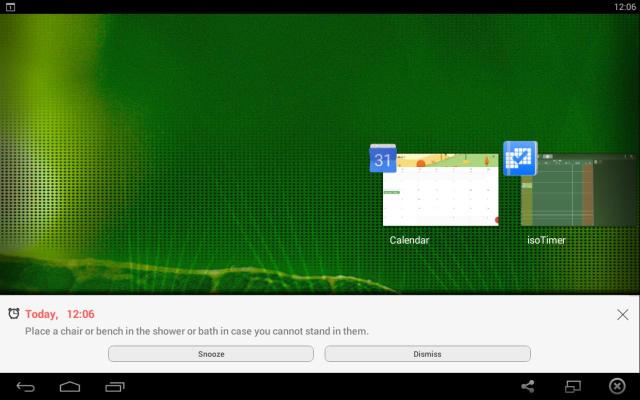
Screenshot of the app isoTimer.

The app used in the study is called isoTimer (see Figure 1) and will be installed on participants’ phones who have a mobile phone with Android OS 3.2 or higher. For correct use of the app, the Google Calendar app will also be installed through which educational advice will be implemented and synchronized on the day and time scheduled by the researcher.

Because of the complications that can occur in this population with the use of mobile phones, the app has been designed to open and start working automatically when the mobile phone is switched on. At no time does the participant have to open the app.

The app works as a reminder, so that the daily educational advice will appear on the screen and the participant must indicate whether the task has been carried out. These responses will be saved in the participants’ mobile phones and then the researcher will download them to be added to the database.

### Ethical Considerations

To carry out this study we will follow the guideline for Good Clinical Practice (GCP) from the International Conference on Harmonisation (ICH), thus guaranteeing protection of the rights, safety, and welfare of trial subjects in accordance with the principles of the Declaration of Helsinki. This will also guarantee the credibility of the clinical trial data.

Before any intervention, each participant and his/her family will be presented with an information sheet and an informed-consent form. This form will explain the study (the voluntary nature thereof), the protection of personal data in accordance with the Organic Law on Personal Data Protection 19/55, as well as their freedom to leave the study at any time they choose.

When the agreement is signed, a copy will be given to each participant, which they will hand in at the clinical trial.

### Outcome Measure

The outcome measure of this study will allow us to know and assess the level of patient dependency, cognitive ability, quality of life, social support, and physical condition. All of these factors will be measured by the Barthel Index, the Functional Independence Measure, the Mini-Mental State Examination, the Canadian Neurological Scale, the Stroke Impact Scale-16, the Trunk Control Test, the Modified Rankin Scale, the Multidimensional Scale of Perceived Social Support, the Quality of Life Scale for Stroke, the Functional Reach Test, the Romberg Test, the Time Up and Go test, the Timed-Stands Test, a portable dynamometer, and a sociodemographic questionnaire (see Table 1).

In addition, visits to the emergency department, the general practitioner, and specialists after discharge from hospital to home, as well as taking drugs associated with stroke, will be controlled.

**Table 1:** Outcome measures of the study.

Test or scale (acronym), reference	Measure	Items, n	Statistical treatment, reference	Variable type
Barthel Index (BI) [39-41]	Level of dependence	10	κw=.93 (95% CI 0.90-0.96) random effects modeling [42,43]	Main
Functional Independence Measure (FIM) [39]	Level of dependence	18	ICC^a^=.124-.661 [44]	Secondary
Mini-Mental State Examination (MMSE) [38]	Cognitive disability	11	ICC=.69 [45]	Secondary
Canadian Neurological Scale (CNS) [46]	Cognitive and motor function	7	κ= 0.76 [47]	Secondary
Stroke Impact Scale-16 (SIS-16) [48]	Quality of life	16	ICC=.70-.92 [49]	Secondary
Trunk Control Test (TCT) [50]	Trunk control	4	ρ=.76, *P*<0.001 [51]	Secondary
Modified Rankin Scale (MRS) [52]	Functional independence	1	Rater 1: κ=.81, .94 and Rater 2: κ=.95, .99 [53]	Secondary
Perceived Social Support Scale (MSPSS) [54,55]	Social support	12	ρ=.72-.85 [54]	Secondary
Quality of Life Scale for Stroke (ECVI-38) [56]	Quality of life	38	ICC=.81-.96 [56]	Secondary
Functional Reach Test (FRT) [57]	Stability	NA^b^	ICC=.90-.95 [58]	Secondary
Romberg Test (RT) [59]	Balance	NA	ICC=.84-.97 [60,61]	Secondary
Time Up and Go (TUG) [62]	Balance, mobility, and fall risk	NA	ICC=.96 [58]	Secondary
Portable dynamometer [63]	Strength in the upper limbs	NA	ICC=.98 [60]	Secondary
Timed-Stands Test (TST) [64]	Strength in the lower limbs	NA	ICC=.994 [65]	Secondary
Sociodemographic questionnaire	Sociodemographic data	25	NA	Secondary

^a^interclass correlation (ICC).

^b^not applicable (NA).

### Procedure

#### Overview

This goal of this study is to implement an educational intervention at home for patients who have suffered a stroke, in order to optimize or improve their performance of ADL after discharge from the hospital to their homes. This intervention will be divided into two stages. In stage 1, assessments will be carried out for the experimental group and the control group, but educational advice will only be provided to the former. Stage 2 comprises a pilot program in which a reminder app will be installed on the mobile phones of some of the participants of the experimental group in order to increase treatment adherence.

#### Stage 1

Stage 1 will begin with the collection of the participants’ demographic data through a questionnaire and by conducting various tests to measure primary and secondary outcome variables. Subsequently, the ergonomics of the home and the implementation of ADL from both the experimental group and the control group will be assessed using the HTAS tool, which was developed by the authors. For the development of the tool, a literature review was performed using the PubMed electronic database and by reviewing different practice guides about stroke. Subsequently, the HTAS tool was evaluated by a panel of experts composed of occupational therapists, physiotherapists, nurses, caregivers, and patients.

Following the assessment of each participant’s home and his or her performance of ADL, the therapist will provide the participants of the experimental group with a list of pieces of advice related to the HTAS tool items that were evaluated negatively. The advice will be aimed at changing the environment in which the participants execute the ADL. This may include facilitation in the execution of the ADL, promoting the active use of the affected side of the body in such execution, or to show them the most appropriate way of performing certain tasks according to their situation after the stroke.

The evaluation of the variables and the execution of the advised tasks will be carried out at participants’ homes 2 and 4 weeks following the initial assessment. Researchers will analyze and compare the data obtained from the outcome variables of the experimental group and the control group to check whether the educational intervention was effective in patients who have suffered stroke and who have been discharged to their homes. If the hypothesis is confirmed, the educational intervention would be implemented in the control group.

#### Stage 2

For the pilot study in stage 2, one group will receive the app reminders on their mobile phones—mobile phone group—and another group will not—no mobile phone group. Placement in the first group depends on whether the participant has a mobile phone and if its characteristics are adapted to the requirements of the study. The app will provide the advice previously given by the therapist in the participants’ homes. The timing of reminders will differ for each participant depending on the amount of advice given. However, in the 4 weeks of the app being used, each piece of advice will be given three times. The mobile phone will beep once for each piece of advice and the participant must check and mark the option indicating whether he has or has not complied with the advice. After this period, the outcome variables will be analyzed in both groups to check whether there are differences between the two groups.

**Figure 2:**
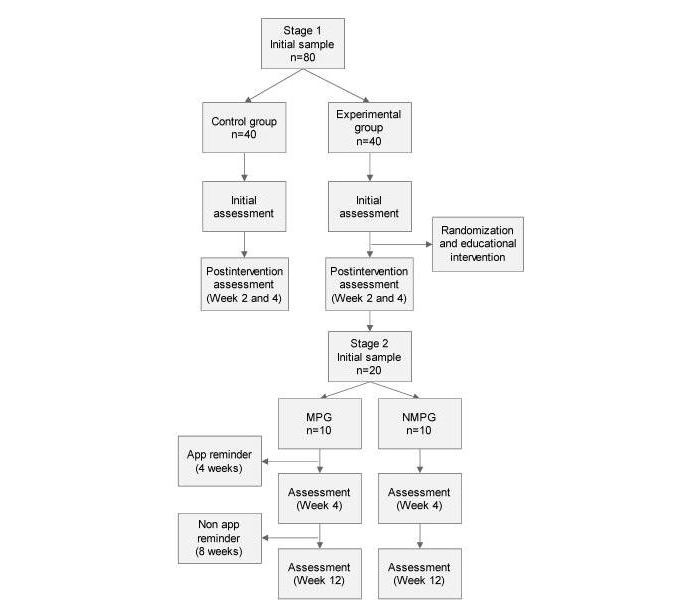
Flowchart of the study protocol. MPG: mobile phone group, NMPG: no mobile phone group.

After 8 weeks, and after having removed the app from mobile phones of the mobile phone group, we will reanalyze the outcome variables. Will do this by testing both groups to see whether the mobile phone group participants have continued to perform the advised tasks provided by the therapist, and if differences still exist between them and the no mobile phone group. If so, a reminder system will be implemented in the no mobile phone group. Figure 2 shows the outline of the entire study protocol, including steps in stage 1 and stage 2.

### Statistical Analyses

Descriptive analyses will include mean, standard deviation, and 95% confidence intervals of the values for each variable. The normality of the variables will be performed using the Kolmogov-Smirnov (KS) test. Preintervention values prior to each condition will be compared. A 2x2 mixed-model analysis of variance (ANOVA) with supplementation (mobile phone group or no mobile phone group) as the between-subjects variable, and time (pre- and postintervention) as the within-subjects variable will be used. Intergroup effect sizes will be calculated (Cohen’s d). An effect size <0.2 reflects a negligible difference, between ≥0.5 and ≤0.8 a moderate difference, and ≥0.8 a large difference. A *P* value <.05 will be considered statistically significant. Data will be analyzed using SPSS version 19.0.

### Sample Size Calculation

A power analysis was conducted using the program G*Power 3.1. A priori, a sample of approximately 40 participants per group for the stage 1 intervention is needed to detect a significant difference (17.3 in the FIM [39]) between the experimental group and the control group (effect size d=0.59, alpha=.05, beta=.08). The randomization will be performed by a blinded researcher.

## Results

Currently, the study is in the recruitment phase and implementation of the intervention has begun. The authors anticipate that during 2015 the following processes should be completed: recruitment, intervention, and data collection. It is expected that the analysis of all data and the first results should be available in early-to-mid 2016.

## Discussion

### Implications of the Study

The aim of this study is that patients who suffer stroke and who have been discharged to their homes improve the performance of ADL in the home environment, in spite of the restrictions caused by the stroke, after advice given by the therapist. A secondary aim is that these patients continue the treatment through a reminder app installed on their mobile phones (isoTimer).

Some studies have analyzed the ergonomics of the workplace through a tool and other studies have carried out different kinds of treatments at home on people with stroke. Because of this, it is thought that an educational intervention at home with people who have suffered a stroke and who have been discharged to their homes could be an innovative and necessary study.

The use of a mobile phone reminder app is an innovative method because the mobile phone has been used with people who have suffered a stroke, but it has not been used before as a means of improving adherence to treatment at home with this population. This method could result in advances in facilitating the continuity of treatment in this population once they return to their homes.

This study could be a breakthrough in the treatment of people who have suffered a stroke and who have been discharged to their homes, since during the first months the largest changes occur and it is essential to continue the rehabilitation treatment received at the hospital. The HTAS tool would allow a proper assessment of the home environment and the implementation of the ADL by the patient, bringing some useful advice that will guide them through the process of recovery of functional independence. Also, being able to have an app that reminds them of the advice provided by the therapist ensures that all patients who suffer a stroke and are discharged to their homes do not encounter barriers in the environment, and that they can advance their functional independence. Additionally, this continuity in the treatment without interruption and its early implementation would favor not only the recovery and rehabilitation of the patient, but could also result in cost savings in care services.

### Conclusions

The aim of this project was that patients who have suffered a stroke and been discharged to their homes can continue with the rehabilitation treatment received at the hospital. This treatment should occur without interruption and in the shortest time possible in order for patients to achieve the highest possible level of functional independence, and so that their readaptation to the environment is optimal. To do this, there will be a therapist intervention using the HTAS tool and an app (isoTimer) that will communicate reminders of the advice. The potential effectiveness of this educational intervention lies in the active participation of the patient.

## Abbreviations

ADL: activities of daily living
ANOVA: analysis of variance
BI: Barthel Index
CNS: Canadian Neurological Scale
ECVI-38: Quality of Life Scale for Stroke
FIM: Functional Independence Measure
FRT: Functional Reach Test
GCP: Good Clinical Practice
HTAS: home therapeutic advice for people who suffer stroke
ICC: interclass correlation
ICH: International Conference on Harmonisation
KS: Kolmogov-Smirnov
MMSE: Mini-Mental State Examination
MRS: Modified Rankin Scale
MSPSS: Perceived Social Support Scale
NA: not applicable
RCT: randomized controlled trial
RT: Romberg Test
SIS-16: Stroke Impact Scale-16
TCT: Trunk Control Test
TST: Timed-Stands Test
TUG: Time Up and Go
